# Dental composite-resin materials and oral-systemic health: clinical toxicology insights

**DOI:** 10.3389/fpubh.2026.1860620

**Published:** 2026-09-22

**Authors:** Wojciech Eliasz, Małgorzata Kujawska, Bennett T. Amaechi, Beata Czarnecka, Elżbieta Paszyńska

**Affiliations:** 1 Department of Integrated Dentistry and Endodontics, Poznan University of Medical Sciences, Poznań, Poland; 2 Department of Toxicology, Poznan University of Medical Sciences, Poznań, Poland; 3 Department of Comprehensive Dentistry, School of Dentistry, University of Texas Health San Antonio, San Antonio, TX, United States; 4 Department of Biomaterials and Experimental Dentistry, Poznan University of Medical Sciences, Poznań, Poland

**Keywords:** dentistry, microplastic, nanoplastic, resin-based composites, toxicology

## Abstract

**Background:**

Resin-based dental composites are the most widely placed direct restorative materials globally. Yet, their organic matrix and complex filler system render them a potential source of biologically active compounds. Residual monomers, including Bisphenol A-glycidyl dimethacrylate (Bis-GMA), triethylene glycol dimethacrylate (TEGDMA), 2-hydroxyethyl methacrylate (HEMA), and urethane dimethacrylate (UDMA), are released into the oral environment through incomplete polymerization, material degradation, and wear, and may subsequently reach systemic circulation. Composite matrix breakdown additionally generates micro- and nanoplastic particles (MNPs), representing a distinct emerging exposure pathway. This review synthesizes clinical and translational evidence on the oral-systemic toxicological implications of resin-based composite materials, with emphasis on biomonitoring data, mechanistic pathways, and relevant risk signals.

**Methods:**

A structured search was conducted across PubMed/MEDLINE, Embase, Scopus, and Web of Science for studies published between January 1990 and September 2025. Eligible studies reported biomonitoring outcomes (saliva, blood, urine, breast milk, cord blood, or placenta), toxicokinetic profiles, or systemic and generalized hypersensitivity reactions attributable to resin-based restorative materials. Studies on dental amalgam and non-dental polymer exposures were excluded. Data were synthesized narratively in accordance with SANRA principles.

**Results:**

Released monomers exert concentration-dependent cytotoxic, genotoxic, and immunomodulatory effects, primarily through glutathione depletion, reactive oxygen species generation, and apoptosis induction. Local adverse effects, including gingival inflammation, pulp cytotoxicity, and allergic contact stomatitis, are well-documented *in vitro* and in clinical case reports. Systemically, BPA and its derivatives have been detected in saliva, urine, blood, breast milk, cord blood, and placenta following dental procedures, raising concerns regarding endocrine-disrupting potential, particularly in children and pregnant women. Dental personnel constitute a distinct occupational risk group through dermal monomer exposure. Composite resins also release MNPs through wear and degradation, with preliminary data suggesting inflammatory and genotoxic potential, though clinical significance remains to be established.

**Conclusion:**

Resin composites pose a measurable, if generally low-level, toxicological risk through monomer elution and, increasingly, MNP release. Adequate operative technique substantially reduces patient exposure. Vulnerable populations, including children, pregnant women, and dental personnel, warrant particular attention. Standardized biomonitoring protocols, updated regulatory thresholds, and dedicated *in vivo* studies are needed to characterize the long-term oral-systemic health implications of these ubiquitous materials.

## 1 Introduction

Dental caries remains one of the most prevalent chronic diseases affecting the global population. According to the Global Oral Health Status Report, approximately 45% of the world’s population is affected by oral diseases, with caries being the most common condition. The World Health Organisation estimates that approximately 2 billion individuals present with caries in their permanent dentition, while an additional 510 million children are affected in the primary dentition (1, 2).

Even though numerous measures have been implemented over the last few decades, numbers show that virtually all humans have been either personally affected or know someone who has experienced consequences of caries (3). The background of the disease, including bacterial factors and lifestyle choices, is well understood, which enabled a gradual shift in the paradigm of treatment from operative to preventive dental treatment (4). However, even the growing popularity of remineralization strategies, including topical fluoride, hydroxyapatite, or bioceramic cements, cannot solve the problem of missing dental hard tissues alone. Once enamel or dentin is lost from the outermost layers of the tooth, it needs to be replaced to allow the oral cavity to perform its functions, including proper mastication, speech, and aesthetics, all of which are crucial for appropriate social interaction (5).

There are several ways to replace missing dental tissues, including direct restorations, indirect reconstructions (crowns or various types of onlays), implant-retained restorations, and dentures. When preventive strategies fail, and dental hard tissue is irreversibly lost, direct restorations are commonly the first line of treatment. Such reconstructions are most commonly performed using composite resin materials or, less commonly, glass-ionomer cements. Bioceramic materials, although they fulfil the ultimate goals of both restorative and preventive dentistry, are often not a feasible option because they lack aesthetics and are unacceptable to a typical dental patient (6). Nevertheless, an emerging compromise has been explored in the form of bioceramic-modified composite resins, including materials in which calcium phosphate compounds (amorphous calcium phosphate, hydroxyapatite), bioactive glass, or calcium silicate particles are incorporated as functional fillers into a conventional resin matrix, with the intent of combining the aesthetic and mechanical advantages of composites with the remineralizing and antibacterial properties of bioceramics However, whether such hybrid materials retain meaningful bioactivity in the complex intraoral environment where ion release is buffered by saliva, competed by adjacent tissues, and rapidly diluted, remains a subject of active debate; clinical evidence is sparse, largely limited to *in vitro* models, and insufficient to establish that the biological benefits observed under controlled laboratory conditions translate into measurable outcomes *in vivo*. A fundamental concern is that the resin matrix itself may act as a diffusion barrier, physically restricting the release of bioactive ions (calcium, phosphate, fluoride) into the surrounding oral fluid and thereby limiting the remineralization potential, which is the primary rationale for their inclusion (6). Although composite resin materials represent the primary option for direct cavity restoration, and such restorations are intended to provide long-term functional and aesthetic rehabilitation, no currently available restorative material meets the strict definition of permanence. All materials are, by nature, subject to degradation, wear, and eventual failure; the clinically relevant question is therefore not whether a restoration will fail, but how long it will remain clinically acceptable.

A more recently recognized, and as yet incompletely characterized, mode of failure involves the emission of microplastics (MPs, <5 mm) and nanoplastics (NPs, <1 μm), collectively termed micro-nano-plastics (MNPs), into the oral cavity and, potentially, the systemic circulation.

Dental composite resin consists of three basic elements: a filler such as silica, a base resin such as Bisphenol A-glycidyl methacrylate (Bis-GMA), and a silane coupling agent that bonds organic and inorganic substances (7). Composite resins have emerged as a source of MNPs among restorative dental materials, due to their complex organic–inorganic structure that combines reacted dimethacrylate monomers, such as (Bis-GMA), Urethane dimethacrylate (UDMA), and Triethylene glycol dimethacrylate (TEGDMA) with nanosized silica or zirconia-based fillers (8). While the primary focus of this review is the elution of residual monomers and additive compounds from composite resins, MNP release is addressed as a secondary and emerging exposure pathway. Given that composite resins are both the most widely used restorative material and a structurally complex polymer-filler system subject to mechanical wear and hydrolytic degradation, the available evidence on MNP generation and its preliminary toxicological signals is synthesized alongside monomer data to provide a comprehensive picture of the oral-systemic risk profile of these materials.

The systemic toxicity of MNPs originating in the oral cavity, particularly those released by dental materials, has become an increasing concern in environmental toxicology and translational medicine (9). However, there is limited direct clinical evidence on the toxicological effects of MNPs from dental composite-resin materials. Therefore, the present article aimed to provide a narrative review of contemporary concepts in dental composite resin materials and to explore their possible connection with systemic health.

Previous reviews regarding the biocompatibility of composite resin materials have mostly addressed monomer cytotoxicity and genotoxicity from the *in vitro* perspective, or have focused bisphenol A release in isolation. What remains insufficiently integrated is the synthesis of human biomonitoring evidence across multiple biological matrices (such as saliva, blood, urine, breast milk, cord blood, and placenta) appraised against the recently revised regulatory thresholds, and, secondly, the emerging and parallel exposure pathway represented by micro- and nanoplastic (MNP) release from the same materials. To the best of our knowledge, neither has any prior review jointly appraised leachate biomonitoring and MNP generation as two aspects of a single source of oral-systemic exposure, nor was the literature re-examined in light of the 2023 EFSA downward revision of BPA tolerable daily intake. Therefore, the present review addresses this gap by stratifying the available evidence explicitly by level and by integrating monomer and MNP data within unified framework.

## 2 Materials and methods

### 2.1 Review design and aim

We present a narrative, evidence-oriented review aiming to synthesize clinical and translational data on the oral-systemic health interface of restorative dental materials, with emphasis on detectable systemic exposure [biomonitoring, toxicokinetic (TK modeling) and clinically relevant toxicological signals]. A narrative design was chosen because the available studies are heterogeneous in design, materials, outcome measures, and analytical techniques, which preclude formal meta-analysis but allow mechanism-aware integration. The present review was conducted as a structured narrative synthesis with a systematic search strategy across the indexed databases.

### 2.2 Information sources and search strategy

A structured search was conducted in PubMed/MEDLINE, Embase, Scopus, and Web of Science from January 1990 to September 2025. The core PubMed strategy was:

((resin-based composite*[tiab] OR “Composite Resins”[Mesh] OR “Dental Adhesives”[Mesh] OR “Dental Cements”[Mesh]) AND (dental[tiab] OR dentistry[tiab] OR dentist*[tiab] OR “dental journals”[sb])) AND (biomonitor*[tiab] OR (“Biological Monitoring”[Mesh]) OR (blood[tiab] OR serum[tiab] OR plasma[tiab] OR urine[tiab] OR “Breast Milk”[Mesh] OR placenta[tiab] OR “Cord Blood”[tiab]) OR toxicokinetic*[tiab] OR pharmacokinetic*[tiab] OR hypersensitivit*[tiab] OR allergy[tiab]) AND (humans[Mesh]) NOT (Amalgam[Mesh] OR “dental amalgam”[tiab]).

This query was adapted to other databases using database-specific subject headings. Reference lists of all eligible papers and key reviews on resin-based materials and BPA/monomer release were hand-searched to identify additional studies.

### 2.3 Eligibility criteria

Inclusion criteria included: (1) Population/setting: human studies in dentistry (patients, children/adolescents, pregnant/lactating women, dental personnel) where restorative dental materials were the exposure; (2) Exposure/materials: direct restorative resin composites, flowables, bulk-fill, compomers, and resin-modified glass ionomer cements, fissure sealants, dental adhesives/bonding systems, and luting cements; (3) Outcomes: any systemic or extra-oral signal linked to these materials, including biomonitoring data (blood/serum/plasma, urine, breast milk, cord blood, placenta), toxicokinetic/pharmacokinetic profiles, reports of systemic or generalized hypersensitivity/allergic reactions, or clinically interpreted toxicology findings; (4) Study design: clinical trials, cohort, case–control, cross-sectional and well-documented case series/case reports providing systemic data; in-vitro or ex-vivo studies were retained only when they provided human-relevant systemic insight (e.g., elution into saliva/serum simulants, TK modeling).

Exclusion criteria included: (1) studies on amalgam/mercury or with amalgam as the primary exposure, (2) non-dental polymer exposures, (3) animal studies without a clear translational/systemic angle, (4) papers without extractable data on systemic exposure or systemic clinical reaction.

### 2.4 Study selection and data extraction

Two reviewers independently screened titles and abstracts retrieved by the search; full texts were obtained for all potentially relevant records. Disagreements were resolved by discussion. A piloted extraction form captured: bibliographic data; country/year; study design; population and age group (incl. Vulnerable groups); dental material (class, brand where reported); clinical procedure (restoration, sealant, adhesive for orthodontic appliances, luting of crowns); exposure descriptor (number/area/age of restorations; time since placement); biomonitoring matrix (saliva, blood, urine, breast milk, cord blood, placenta), analytical method (e.g., LC–MS/MS, GC–MS, HPLC), analyte (BPA, Bis-GMA, TEGDMA, UDMA, other monomers/additives), concentration and time-course, and reported clinical/toxicology outcomes (hypersensitivity, systemic symptoms, endocrine-relevant findings). Where studies provided only maximum or peak post-treatment values, these were extracted as such.

### 2.5 Synthesis

Because of heterogeneity in materials, analytical methods and reporting units, data were synthesized narratively. Studies were first grouped by whether they addressed local or systemic exposure and further by the research model, i.e., *in vitro*/*ex vivo* vs. clinical trials.

### 2.6 Assessment of rigor

Methodological quality was appraised pragmatically: for clinical/observational studies, we noted sampling schedule vs. expected half-life, use of blanks/contamination controls, adjustment for background BPA/methacrylate exposure (diet, environment), and clarity regarding dental exposure. The risk of selection bias inherent to the narrative format was mitigated through a pre-specified multi-database search strategy, broad inclusion criteria, and supplementary citation tracking.

## 3 Results

In line with the aim of the review, the evidence is organized by level. Findings derived from *in vitro* and *ex vivo* models, animal models, and human clinical and biomonitoring studies are distinguished explicitly, because their clinical significance differs to a significant extent, and laboratory findings cannot be extrapolated directly to clinical situations.

Even though resin-based dental composites provide excellent esthetics and mechanical properties relevant to many dental situations, they may inherently pose biological safety concerns due to the release of unbound monomers and additives resulting from incomplete or improper polymerization, material degradation, or wear over time (10). Major concerns relate to the elution of Bisphenol A-glycidyl methacrylate (Bis-GMA), Triethylene glycol dimethacrylate (TEGDMA), 2-hydroxyethyl methacrylate (HEMA), and Urethane dimethacrylate (UDMA), as well as contaminants such as Bisphenol A (BPA) (11–16). The mechanisms underlying the adverse biological effects of resin-based composite materials are summarized in Figure 1. It illustrates the comprehensive toxicological pathways associated with resin-based dental composites, tracing the progression from initial material degradation to distinct clinical outcomes. The flowchart shows two primary modes of matrix breakdown: the elution of unreacted monomers (e.g., Bis-GMA, TEGDMA, HEMA) due to incomplete polymerization, and the emerging pathway of micro- and nanoplastic (MNPs) generation via mechanical wear and hydrolysis.

**Figure 1:**
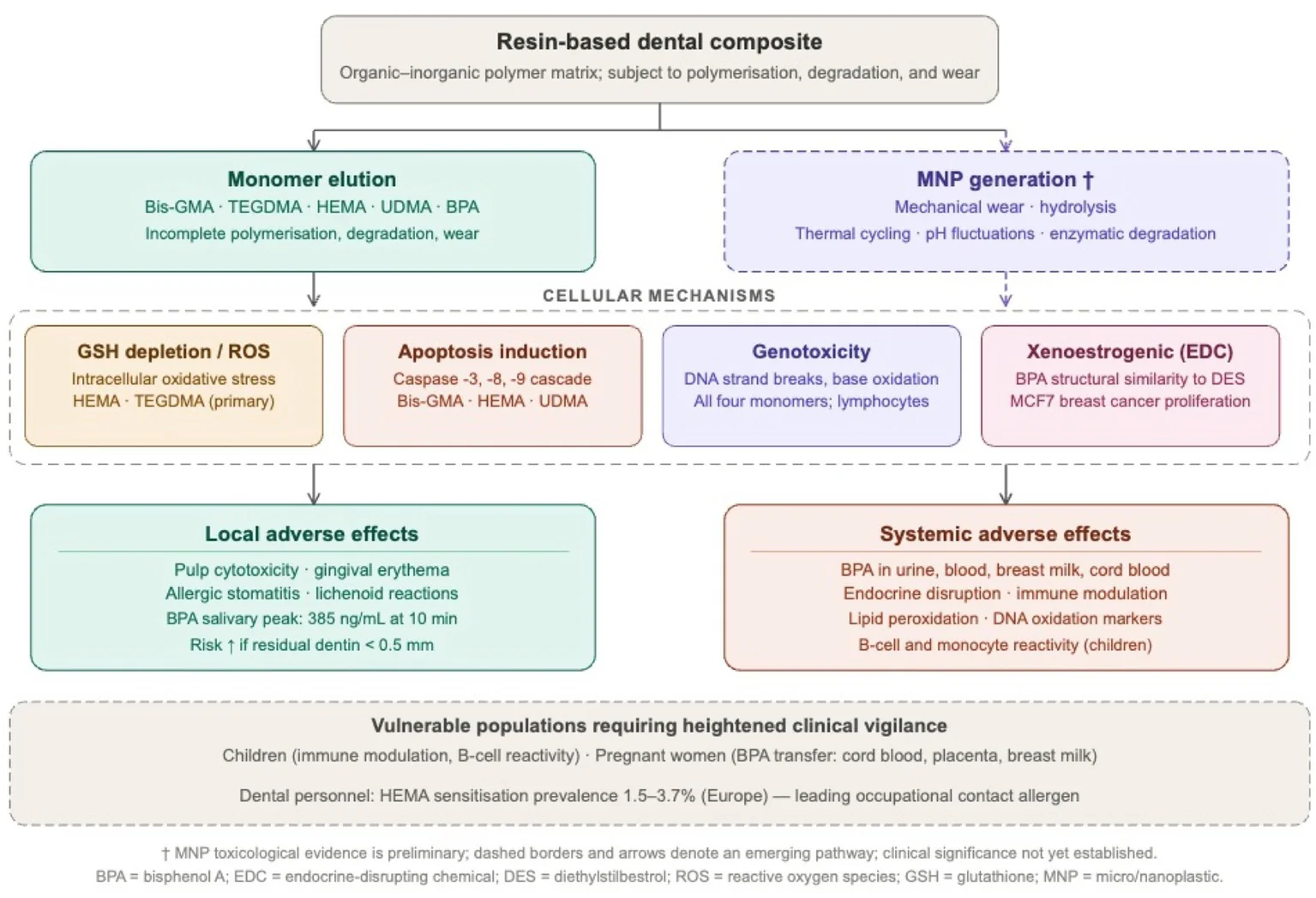
Comprehensive toxicological pathways associated with resin-based dental composites, tracing the progression from initial material degradation to distinct clinical outcomes.

### 3.1 Local adverse effects

Local adverse effects are those that occur directly at or near the site of application and affect oral tissues such as the dental pulp, gingiva, and oral mucosa (16). At the *in vitro* and *ex vivo* level, the majority of early investigations relied on laboratory cell-culture models, whose findings are mechanistic and cannot be extrapolated directly to patients, however, they provided findings used later to frame the issue of material toxicity.

#### 3.1.1 *In vitro* and *ex vivo* evidence

The starting point for the majority of investigations relied on *in vitro* studies. Such investigations employed cell cultures derived from oral tissues, such as primary human gingival fibroblasts (HGFs), human pulpal fibroblasts, murine macrophages, and immortalized mouse fibroblasts (3T3, L929), have established the cytotoxic potential of released monomers (17–21). Monomers such as TEGDMA, HEMA, and Bis-GMA are known to diffuse through dentin into the pulp space and directly contact gingival tissues, leading to cytotoxic effects. Cytotoxicity varies significantly among monomers, with some sources suggesting a generalized ranking of toxicity based on concentration required for effect: Bis-GMA > UDMA > TEGDMA (11). The substantial release of TEGDMA and HEMA is of particular biological concern, as both monomers have been associated with cytotoxic, genotoxic, and pro-inflammatory effects (17). Furthermore, uncured bonding agents have been shown to exert greater cytotoxicity toward odontoblast-like cells compared to their polymerized counterparts, underscoring the clinical relevance of complete monomer conversion, as unpolymerized one-step dentin-bonding agents have been shown to exert greater cytotoxicity toward both human dental pulp cells and odontoblast-like cells (MDPC-23) than their polymerized counterparts, with cell viability reduced by 2–65% following complete photopolymerization (22, 23).

The choice of the cell models in such studies reflects a compromise between clinical relevance and experimental standardization. Primary human gingival fibroblasts and dental pulp cells are used because they represent the tissues most directly exposed to eluted monomers at the restoration site, whereas established murine lines such as 3 T3 and L929 fibroblasts are internationally standardized reference cell recommended for cytotoxicity screening (ISO 10993-5), offering a reproducible and comparable results across laboratories (24). Odontoblast-like cells are employed as a surrogate for the odontoblastic layer that forms the first cellular barrier beneath dentin. These models allow controlled dose–response testing that is impossible in patients but what they lack is the influence of actual pulpal blood flow, buffering capacity of dentin, and clearance resulting from exposure to oral fluids, which all results in absolute concentrations not being translatable to clinical settings.

Several mechanisms were linked to potential toxic pathways, including cellular pathway activation and genotoxic effects. Cellular pathways associated with the influence of materials on vital tissues include glutathione (GSH) Depletion and ROS generation. HEMA and TEGDMA exposure lead to depletion of intracellular GSH. GSH depletion impairs the cell’s protective capacity against toxicants, particularly reactive oxygen species (ROS) (14). HEMA is specifically known to stimulate GSH depletion and ROS production in dental pulp cells and gingival epithelial cells (24, 25). Non-irradiated camphorquinone (CQ), a photoinitiator, was also shown to induce DNA damage in HGFs starting at low concentrations (0.05 mmol/L) (26). Furthermore, toxicity may be expressed as the potential to induce apoptosis and necrosis. Among these, HEMA is a potent inducer of apoptosis in human and mouse cells, particularly through ROS formation, whereas Bis-GMA induces cytotoxicity, including apoptosis and necrosis, through activation of caspases-3, −8, and −9 in murine macrophages (25–27).

On the other hand, composite materials may induce genotoxic effects, as shown for HEMA, TEGDMA, UDMA, and Bis-GMA in human oral cells and lymphocytes (20, 26, 28). Bis-GMA determines genotoxic effect through oxidative modifications of DNA bases and DNA double-strand breaks, which was observed in human salivary gland tissue cells and lymphocytes exposed to these methacrylates, suggesting a tumor-initiating potency (28).

Even though dentin is now seen as a biological barrier that can mitigate potential toxicity due to its buffer capacity, experiments using dentin barriers showed that components released from materials may, in fact, diffuse into the pulp tissue through dentinal tubules. If the remaining dentin thickness is below 0.5 mm, a strong correlation with potential inflammation has been reported (19).

#### 3.1.2 Clinical evidence

Apart from *in vitro* and *ex vivo* examinations, clinical reports focused on visible or measurable local adverse responses, particularly monomer release into oral fluid and tissue reactions, were also published. They investigated such factors as Salivary Monomer Release (29). As released monomers enter the oral environment (saliva), this results in patient exposure. Studies have quantified BPA, Bis-GMA, and its methacrylate derivative in human saliva following placement of composite restorations or sealants. A study examining BPA release after treatment reported a high mean concentration of 385 ng/mL in saliva 10 min after treatment, which rapidly decreased to 88.2 ng/mL after 1 h and 1.85 ng/mL after 24 h (30). HEMA and TEGDMA are available in microgram quantities via the salivary surface in the minutes and hours after clinical placement (21).

The examination of oral adverse reactions constitutes another important part of gaining an insight into the topic. Local adverse effects are rare but reported reactions linked to residual monomers include allergic contact stomatitis, mild erythema in the gingiva and buccal mucosa, burning mouth syndrome, and lichenoid-like reactions of the lips and oral mucosa (31).

### 3.2 Systemic adverse effects

Systemic effects refer to the potential for eluted compounds to reach the bloodstream and affect organs beyond the mouth, and the evidence comes from three levels: laboratory models, human biomonitoring, and, more recently, studies of particulate release.

#### 3.2.1 *In vitro* and *ex vivo* evidence

For the purpose of investigating the systemic effects, studies rely mostly on non-oral human cell lines and cell-free oestrogeniciy assays as proxies for distant organ and endocrine effects. Human peripheral blood lymphocytes and mononuclear cells are used to model geno- and immunotoxic potential in circulating cells, whereas hepatic systems are used because of the liver being the principal site of monomer metabolism, and oestrogen-responsive MCF-7 breast cancer lines is a conventional model for detecting xenoestrogenic activity (32).

Systemic effects concern the potential for released compounds to enter the bloodstream and influence distant organs or systems, often evaluated by measuring biomarkers in urine or blood, or testing effects on non-oral cell types. Systemic toxicity studies frequently utilize non-oral cell lines, such as human peripheral blood lymphocytes, liver, or lung cells, to assess broader biological impacts, particularly genotoxicity and endocrine disruption. Monomers induce cytotoxicity and apoptosis in human peripheral blood mononuclear cells (PBMCs). For example, UDMA decreased mitochondrial activity in PBMCs by 50–93% at certain concentrations (33, 34). The dental composite component TEGDMA and its metabolic by-products were studied in human pulmonary A549 cells (15). The metabolite 2,3-epoxymethacrylic acid (2,3-EMA) showed cytotoxicity comparable to or greater than that of the parent TEGDMA in A549 cells. Genotoxic effects induced by methacrylates such as HEMA, TEGDMA, Bis-GMA, and UDMA in peripheral lymphocytes are well documented (28, 35, 36). Glycidyl methacrylate (GMA) evoked concentration-dependent DNA damage in human lymphocytes (36). Components from composites and adhesives can diffuse through dentin into the pulp, becoming bioavailable for metabolism. Epoxides, such as 2,3-EMA, formed by the metabolism of TEGDMA and HEMA in hepatic microsomes, are regarded as highly reactive, potentially mutagenic, and carcinogenic agents.

Another important issue concerns the release of BPA. Bisphenol A (BPA) and its derivatives are known endocrine-disrupting chemicals (EDCs). Due to its structural similarity to diethylstilbestrol (DES), BPA is thought to have xenoestrogenic effects (37–40). Low concentrations of BPA (and its derivatives) were observed to stimulate the proliferation of MCF7 human breast cancer cells, as did estradiol-17b (41). The lowest observed effect concentrations for BPA phenols in *in vitro* assays ranged from 100 to 1,000 nM (39).

The toxicity issue was also evaluated in clinical studies that typically involved analyzing body fluids (urine, blood) for the presence of monomers or their metabolites. Monomers released into the oral cavity can be absorbed locally or swallowed with saliva, potentially reaching the bloodstream and inducing a systemic adverse response (12, 13, 42). Detectable levels of free monomers and potential toxic exposure have been reported in saliva, urine, and blood samples following restoration. BPA was quantified in human urine after dental treatment. One study reported a mean pre-treatment urinary BPA concentration of 1.41 ng/mL, with levels reaching 1.99 ng/mL 1 week after treatment (though this rise was not statistically significant compared to pre-treatment) (30). Another study reported creatinine-adjusted urinary concentrations of BPA peaking 1 h after sealant placement (mean 20.1 micrograms/g creatinine) (43). Table 1 summarizes key human studies reporting measured concentrations of BPA and structural monomers, including Bis-GMA, TEGDMA, HEMA, and UDMA, in saliva, urine, and blood before and after placement of composite restorations or dental sealants, together with the associated clinical and biological findings.

**Table 1:** Summary of key *in vivo* studies assessing systemic exposure and the systemic effects of resin-based composite materials.

Study/year	Study population	Biomarker measured (matrix)	Key results and concentration changes	Clinical relevance
Berge et al. (2019) (30)	20 patients (16–40 years) receiving composite restorations	BPA (Urine and saliva)	A significant, exponential increase in salivary BPA concentration was noted immediately post-treatment, with a marginal elevation at 1 week. No significant changes were detected in urine.	The highest systemic exposure to BPA is acute, transient, and largely limited to the period immediately following application.
Joskow et al. (2006) (43)	14 adult patients receiving dental sealants	BPA (Urine and saliva)	BPA levels rose sharply in saliva, and remained elevated in urine for 24 h after the application of the Delton LC sealant.	Estimated acute BPA doses in humans can reach levels that induced estrogen-receptor-mediated effects in animal models. However, salivary collection post-treatment can reduce systemic absorption.
Gul et al. (2021) (47)	18 healthy males receiving new composite fillings	BPA (Blood serum and saliva) and reproductive hormones	Serum BPA levels were higher than in saliva (from 1.01 to 1.49 ng/mL), but did not significantly increase above baseline values over a 5-week observation.	Demonstrated that BPA concentrations released into the bloodstream after composite placement do not reach toxic doses and do not disrupt systemic levels of male reproductive hormones.
Maserejian (2014) (46)	Children monitored over a 5-year post-intervention period (NECAT study)	Immune system cells and markers (Peripheral blood)	Changes in overall white blood cell count, T-cell, and neutrophil activity were marginal. Increased B-cell reactivity correlated with a higher number of Bis-GMA-based restorations.	Despite lacking evident immunological deficits, this proved that elevated exposure to methacrylate polymers in children may have a certain systemic modulating impact on B-lymphocyte activation in the bloodstream.
Di Pietro et al. (2008) (48)	Adolescents and adults undergoing routine conservative treatment	DNA damage (Peripheral blood lymphocytes)	An increase in the level of DNA damage was observed in peripheral blood mononuclear cells of subjects with composite dental fillings compared to the control group.	This study provides direct evidence of the systemic genotoxic potential of released resin monomers, whose mechanism in humans is based on oxidative stress.

#### 3.2.2 Toxicokinetic interpretation of biomonitoring data

Accurate interpretation of biomonitoring findings requires that the underlying toxicokinetics of leached compounds, particularly BPA should be taken into account. Its biological half-life is approximately 6 h, with elimination nearly complete within 24 h. BPA exposure from dental materials constitutes rather an acute, transient event, which results in the salivary and urinary concentrations spiking within the first hours after restoration placement, then declining exponentially, returning to near-baseline levels within 24 h. While the material itself continues to release trace amounts of BPA over subsequent weeks to months at progressively diminishing rates, rapid metabolic clearance prevents sustained accumulation. Dental composites and sealants therefore represent time-constrained source of exposure rather than a continuous contributing factor for systemic retention (30, 43).

Despite the fact that these spikes are short in duration, their clinical significance cannot be fully dismissed. Unlike classical toxic molecules, EDCs such as BPA frequently exhibit non-monotonic dose–response relationships displaying biological effects at lower concentrations that are not observed at higher doses (44). Accordingly, although total leached doses remain well below established TDI values, the non-monotonic nature of BPA reinforces the rationale for simple clinical procedures whose aim is to rinse out potential toxic molecules out of the oral cavity (rinsing, appropriate finishing, isolation).

Nevertheless, systemic toxicity is generally considered low because the quantities of leachable chemicals released are small and tend to decrease within a relatively short time after restoration placement (21, 45). However, components can be ingested and absorbed via the gastrointestinal mucosa. In children, alterations in immune responses (changes in B-cell reactivity and a decline in monocyte reactivity) were reported following resin-matrix composite restoration, specifically pronounced with higher Bis-GMA exposure (46). Increased markers of lipid peroxidation and DNA oxidation were observed in participants with resin-matrix composite fillings, attributed to Bis-GMA and TEGDMA released into the oral cavity and subsequently monitored through blood circulation (47). Genotoxicity has been assessed via biomonitoring of DNA damage in peripheral blood lymphocytes in subjects with dental restorative fillings (48). Of particular concern is 2-hydroxyethyl methacrylate (HEMA), the single most prevalent monomer in acrylate-based dental and cosmetic products, identified in 58% of nail cosmetics surveyed and in the majority of dental bonding systems, which is also consistently the most frequently patch test-positive methacrylate, with sensitisation prevalences of 1.5–3.7% in Europe and 3.2% in North America, and a disproportionate impact on dental personnel among whom it represents the leading occupational allergen (49).

#### 3.2.3 Micro- and nanoplastics (MNPs)

The application of polymer-based biomaterials in dentistry has inadvertently contributed to contamination of clinical environments and the oral cavity with MNPs. MNPs in dentistry may originate from mechanical wear, chemical degradation, or thermal and pH-induced polymer fragmentation (50–53). Some risks might be associated with the oral-systemic health from the MNPs contamination as a result of their physical properties or their chemical compositions (54). MNPs are bioavailable, able to cross epithelial barriers, and are distributed throughout the body, potentially accumulating in tissues and causing multi-organ toxicity (55–57). Although these materials are extensively used in conservative and restorative dentistry due to their mechanical and aesthetic properties, in the oral environment, these materials may undergo degradation and release MNPs, exposing both patients and dental professionals to potential risk (9). Furthermore, mechanical forces, enzymatic action of salivary esterases, pH fluctuations, thermal cycling, and procedural abrasion can break down these composite materials, releasing micro- and nanosized particles into the oral environment. The risks associated with oral health are connected to the relatively under-researched oral exposure route via the ingestion and inhalation of MNPs derived from dental materials and oral-care products which has been associated with oral mucosal irritation, dysbiosis in the oral cavity, genotoxic effects on oral cells, and potential carcinogenicity (58–60). Salivary studies further support the presence and activity of MNPs in the oral cavity (61, 62). Even though its potential influence on the oral cavity is becoming more frequently addressed in literature, direct *in vivo* evidence from dental sources remains extremely limited.

## 4 Discussion

Careful examination of the provided data suggests that the body of evidence is uneven, when the level and strength are taken into account. The most abundant data derive from *in vitro* and *ex vivo* models, which consistently demonstrate that the effects, both cyto- and genotoxic, of released monomers are concentration-dependent (15, 17, 18, 20). Nevertheless, these models typically employ concentration that significantly exceed physiological levels, frequently in static condition, in which salivary turnover, mucosal clearance, and metabolic processes are omitted. Human evidence is comparatively sparse and is dominated by short-term biomonitoring rather than clinical outcome data. Consequently, the strength of the evidence may be graded as high for the transient systematic detectability of leeched compounds, moderate for concentration-dependent *in vitro* toxicity, and low for clinically meaningful systemic harm at realistic exposure levels.

A distinction should also be drawn between intrinsic monomer potency and effective clinical exposure. Although a generalized cytotoxicity raking of Bis-GMA being the most toxic, and TEGDMA the least toxic based solely on concentration basis (10), TEGDMA and HEMA are of much greater practical concern because of their higher hydrophilicity, and their ability to diffuse through barriers, and easier elution into the aqueous oral environment (12, 21). Therefore, the priorities may influence the ranking, as data regarding intrinsic potency should be discussed along data regarding realistic exposure values.

Such juxtaposition may also prove worthwhile when discussing discrepancies in internally inconsistent data found in the human biomonitoring literature, as differences in study methodology may account for the majority of such outcomes. Divergent urinary BPA outcome with marked salivary spikes but no significant urinary change in some studies, and sustained urinary elevation in others, are consistent once, e.g., sampling schedules are considered against the biological half-life of BPA, which is approximated at 6 h (30, 43, 63–66). When this is taken into account, studies sampling beyond the acute post-operative window will predictably report null results. Similarly, the contrast between negligible disruption of male reproductive hormones in one cohort, and exposure estimates reaching levels that are relevant for oestrogen receptor, may reflect differences in analytical matrix (saliva vs. serum or urine), adjustment for background dietary and environmental BPA, and should also be nuanced against the data regarding the actual influence of concentration and biological effect. This underscored the need for standardized sampling and reporting in future studies.

However, the release of molecules such as BPA from dental composites must be compared with the established regulatory benchmarks. The 2015 EFSA Tolerable Daily Intake (TDI) of 4 μg/kg body weight/day places standard composite restorations in a highly favorable safety margin: pharmacokinetic modeling indicates median BPA exposure per dental procedure is typically below 10 ng, approximately 4,000-fold beneath this threshold. Even worst-case scenarios involving unpolymerized materials (approximately 533 ng per treatment) remain well within safe limits. Nevertheless, the regulatory context has shifted substantially. In 2023, EFSA revised the TDI downward by several orders of magnitude to 0.2 ng/kg body weight/day, which is a threshold already exceeded by dietary exposure across all age groups. Illustrative calculations indicate that Bis-GMA-containing materials contribute 13–33% to the revised daily TDI in a 20 kg child, while most composites contribute less than 3%, under conservative assumptions that do not include the effect of saliva turnover or mucosal clearance (67). Given the recent tightening of regulatory limits and the potential health risks associated with even low-dose BPA exposure, a re-evaluation of their impact is necessary. Further research with standardized methodologies is essential to accurately quantify BPA release and its implications for patient safety (68).

These findings translate into several practical measures whose aim would be to reduce residual monomer and particle exposure. Because salivary concentrations peak within minutes of placement and decline sharply afterwards, post-placement rinsing and brief delay in drinking or eating are the simplest means of reducing the ingested dose (30, 69). Adequate polymerization is the single most important operator-dependent of monomer release, as unpolymerized resins, which included adhesive agents in the cited studies, are markedly more cytotoxic than their cured counterparts. Therefore, paying adequate attention to curing time, energy density, and distance between the tip of the polymerizing unit is essential (12). Also, immediate finishing procedures remove the incompletely polymerized surface layer, known as the oxygen inhibition layer, that constitutes the primary source of monomers. Also, effective isolation, ideally with rubber dam, may limit salivary contact and uptake of eluted monomer (70). Placement of a BPA-derivative-free or documented low-elution materials or systems, may constitute a reasonable precaution of vulnerable populations, such as children and pregnant or lactating women (71). However, it should be underlined that these recommendations are based on mechanistic and elution data rather than on long-term clinical outcome trials, and are best framed as low-burden precautions.

Several limitations should be applied when discussing the outcomes of the review. First of all, the body of evidence is dominated by *in vitro* studies using concentrations that have not been observed in physiological conditions, and static models that omit the complex factors present in the oral cavity, such as salivary clearance, mucosal turnover, or metabolic factors. This limits their extrapolation potential to the clinical setting. Secondly, human studies are heterogenous in material, exposure measures (number of restorations, surface area of restorations), sampling schedule, analytical platforms, or adjustment for background environmental and dietary exposure, which precludes appropriate pooling. The third issue is related to the lack of long-term prospective clinical outcome oriented data, as the available studies capture the short-term biomonitoring picture rather than health endpoints. Fourth, evidence on MNP release from dental composites remains preliminary and scarce, with negligible direct *in vivo* human data. Lastly, as a narrative review, the present synthesis is subject to selection and interpretive bias, mitigated but not eliminated by the pre-specified multi-database search and supplementary citation tracking.

Despite this growing mechanistic evidence, the field remains constrained by several important limitations. Direct *in vivo* and clinical exposure data are still sparse, and detection and quantification of MNPs in biological or environmental dental samples remain technically demanding. Establishing reliable methods to identify and measure MP/NP particles in complex biological matrices continues to represent a major methodological obstacle. Although *in vitro* studies demonstrate measurable particle release from common dental materials, real-world exposure data remain limited and inconsistent and no validated toxicokinetic framework exists for particulate MNPs comparable to those available for small-molecule dental monomers (72, 73).

Acknowledging these limitations may, however, point to the priority areas for future studies. First of all, long-term prospective clinical cohorts linking composite exposure to health endpoints should be included. Moreover, standardized and harmonized toxicity and elution testing protocols would enable easier and valid cross-study comparison. A systematic evaluation of newer, BPA-free and low-elution systems or protocols under realistic clinical conditions constitutes another interesting direction, along with the development and validation of bioactive, low-leachate restorative materials that reconcile mechanical and aesthetic performance with a reduced biological burden.

## 5 Conclusion

According to presented data, resin composites release residual monomers and, increasingly recognized, micro- and nanoplastic particles that are detectable within the oral cavity in systemic compartments. The available evidence robustly demonstrates transient systemic detectability and concentration-dependent *in vitro* toxicity, but does not establish clinically meaningful systemic harm at realistic exposure levels. Operative technique is a modifiable determinant of exposure, and vulnerable populations, including children, pregnant women, and dental personnel, warrant particular attention. In light of the 2023 downward revision of the bisphenol A tolerable daily intake and the emerging interest in micro- and nanoplastic exposure pathway, standardized biomonitoring, unified toxicity testing, and long-term prospective clinical studies are needed to define the true oral-systemic risk of these ubiquitous materials. From a public health perspective, the population-level implications of these findings warrant particular emphasis, as resin composites are placed in billions of teeth worldwide and across the entire life course, even low-level individual exposures aggregate into a cumulative population burden, and the groups most affected, such as children, pregnant and lactating women, and dental personnel, are precisely those in whom preventive public health measures yield the greatest benefit. Embedding exposure-minimizing clinical protocols into routine practice, alongside regulatory standards and biomonitoring frameworks calibrated to cumulative rather than single-event exposure, therefore represents a pragmatic, prevention-oriented contribution to population oral and systemic health.
